# Association of Cancer Diagnosis and Therapeutic Stage With Mortality in Pediatric Patients With COVID-19, Prospective Multicenter Cohort Study From Latin America

**DOI:** 10.3389/fped.2022.885633

**Published:** 2022-05-03

**Authors:** Jesus Ángel Dominguez-Rojas, Pablo Vásquez-Hoyos, Rodrigo Pérez-Morales, Ana María Monsalve-Quintero, Lupe Mora-Robles, Alejandro Diaz-Diaz, Silvio Fabio Torres, Ángel Castro-Dajer, Lizeth Yuliana Cabanillas-Burgos, Vladimir Aguilera-Avendaño, Edwin Mauricio Cantillano-Quintero, Anna Camporesi, Asya Agulnik, Sheena Mukkada, Giancarlo Alvarado-Gamarra, Ninoska Rojas-Soto, Ana Luisa Mendieta-Zevallos, Mariela Violeta Tello-Pezo, Liliana Vásquez-Ponce, Rubén Eduardo Lasso-Palomino, María Camila Pérez-Arroyave, Mónica Trujillo-Honeysberg, Juan Gonzalo Mesa-Monsalve, Carlos Alberto Pardo González, Juan Francisco López Cubillos, Sebastián Gonzalez-Dambrauskas, Alvaro Coronado-Munoz

**Affiliations:** ^1^Pediatric Critical Care, Hospital Edgardo Rebagliati Martins, Red Colaborativa Pediátrica de Latinoamérica (LARed Network), Lima, Peru; ^2^Pediatric Critical Care, Hospital de San Jose, Red Colaborativa Pediátrica de Latinoamérica (LARed Network), Bogota, Colombia; ^3^Research Division, Department of Pediatrics, Fundacion Universitatia de Ciencias de la Salud–FUCS, Bogota, Colombia; ^4^Pediatric Critical Care, HOMI Fundacion Hospital Pediatrico La Misericordia, Bogota, Colombia; ^5^Pediatric Critical Care, Fundación Valle del Lili, Cali, Colombia; ^6^Pediatric Critical Care, Hospital SOLCA, Cuenca, Ecuador; ^7^Pediatric Infectious Diseases, Hospital Pablo Tobon Uribe y Hospital General de Medellin, Medellin, Colombia; ^8^Pediatric Critical Care, Hospital Universitario Austral Pilar, Buenos Aires, Argentina; ^9^Pediatric Oncology, Clinica Blas de Lezo, Cartagena, Colombia; ^10^Deparment of Pediatrics, Hospital Nacional Carlos Alberto Seguin Escobedo Essalud, Arequipa, Peru; ^11^Pediatric Critical Care, Hospital del Niño Doctor Ovidio Aliaga Uría, La Paz, Bolivia; ^12^Pediatric Critical Care, Hospital del Norte, San Pedro Sula, Honduras; ^13^Department of Pediatric Anesthesia and Intensive Care, Vittore Buzzi Children's Hospital, Milano, Italy; ^14^Department of Global Pediatric Medicine, St. Jude Children's Research Hospital, Memphis, TN, United States; ^15^Pediatrics, Hospital Edgardo Rebagliati Martins, Lima, Peru; ^16^Instituto de Investigación Nutricional, Lima, Perú; ^17^Hematóloga Pediatra, Hospital Edgardo Rebagliati Martins, Lima, Peru; ^18^Pediatric Emergency Department, Hospital Edgardo Rebagliati Martins, Lima, Peru; ^19^Pediatric Oncology, Instituto Nacional de Salud del Niño, San Borja, Perú; ^20^Research Center “Medicina de Precisión, ” Facultad de Medicina, Universidad de San Martín de Porres, Lima, Perú; ^21^Pediatric Infectious Disease, Hospital Pablo Tobon Uribe, Medellín, Colombia; ^22^Infectólogo Pediatra, Hospital General de Medellin, Medellin, Colombia; ^23^Hemato-oncólogo Pediatra, HOMI Fundación Hospital Pediátrico La Misericordia, Bogotá, Colombia; ^24^Infectólogo Pediatra, HOMI Fundación Hospital Pediátrico La Misericordia, Bogota, Colombia; ^25^Specialized Pediatric Critical Care (CIPe), Casa de Galicia, Red Colaborativa Pediátrica de Latinoamérica (LARed Network), Montevideo, Uruguay; ^26^Medical School, Pediatric Critical Care, Pereira Rossell Medical Center (UCIN-CHPR), Universidad de la República, Montevideo, Uruguay; ^27^Pediatric Critical Care Division, Department of Pediatrics, University of Texas Health Science Center at Houston, Houston, TX, United States

**Keywords:** pediatric cancer, pediatric, COVID-19, child development, PICU (pediatric intensive care unit)

## Abstract

**Background:**

Children with cancer are at risk of critical disease and mortality from COVID-19 infection. In this study, we describe the clinical characteristics of pediatric patients with cancer and COVID-19 from multiple Latin American centers and risk factors associated with mortality in this population.

**Methods:**

This study is a multicenter, prospective cohort study conducted at 12 hospitals from 6 Latin American countries (Argentina, Bolivia, Colombia, Ecuador, Honduras and Peru) from April to November 2021. Patients younger than 14 years of age that had an oncological diagnosis and COVID-19 or multisystemic inflammatory syndrome in children (MIS-C) who were treated in the inpatient setting were included. The primary exposure was the diagnosis and treatment status, and the primary outcome was mortality. We defined “new diagnosis” as patients with no previous diagnosis of cancer, “established diagnosis” as patients with cancer and ongoing treatment and “relapse” as patients with cancer and ongoing treatment that had a prior cancer-free period. A frequentist analysis was performed including a multivariate logistic regression for mortality.

**Results:**

Two hundred and ten patients were included in the study; 30 (14%) died during the study period and 67% of patients who died were admitted to critical care. Demographics were similar in survivors and non-survivors. Patients with low weight for age (<-2SD) had higher mortality (28 vs. 3%, *p* = 0.019). There was statistically significant difference of mortality between patients with new diagnosis (36.7%), established diagnosis (1.4%) and relapse (60%), (*p* <0.001). Most patients had hematological cancers (69%) and they had higher mortality (18%) compared to solid tumors (6%, *p*= 0.032). Patients with concomitant bacterial infections had higher mortality (40%, *p* = 0.001). MIS-C, respiratory distress, cardiovascular symptoms, altered mental status and acute kidney injury on admission were associated with higher mortality. Acidosis, hypoxemia, lymphocytosis, severe neutropenia, anemia and thrombocytopenia on admission were also associated with mortality. A multivariate logistic regression showed risk factors associated with mortality: concomitant bacterial infection OR 3 95%CI (1.1–8.5), respiratory symptoms OR 5.7 95%CI (1.7–19.4), cardiovascular OR 5.2 95%CI (1.2–14.2), new cancer diagnosis OR 12 95%CI (1.3–102) and relapse OR 25 95%CI (2.9–214).

**Conclusion:**

Our study shows that pediatric patients with new onset diagnosis of cancer and patients with relapse have higher odds of all-cause mortality in the setting of COVID-19. This information would help develop an early identification of patients with cancer and COVID-19 with higher risk of mortality.

## Background

The mortality associated with COVID-19 in pediatric patients has been lower compared to adults (1–3). However, pediatric patients with comorbidities such as obesity and immunosuppression are at higher risk of morbidity and mortality than healthy patients (4). A population that has been considered at risk are children with cancer.

At the beginning of the pandemic multiple studies from first-line providers and pediatric oncologists from different countries, reported their experience in children with cancer and COVID-19, and shared their concerns regarding possible complications in this population (5–16). Some studies also described the impact of the pandemic on patient care and resulting delays in diagnosis for oncological patients (17–20).

Identification of factors associated with mortality in childhood cancer patients diagnosed with COVID-19 could help providers optimize management of these patients. Factors of interest include the primary oncology diagnosis, the diagnostic stage (new diagnosis vs.stable disease vs. relapse), the stage of treatment (treatment-naïve vs. active treatment), and treatment intensity and their complications. These factors could be associated with higher mortality and critical COVID-19 presentation, leading to prolonged hospitalization, multisystem failure, and prolonged mechanical ventilation. An important study regarding outcomes in this population is the “Global characteristics and outcomes of SARS-CoV-2 infection in children and adolescents with cancer (GRCCC)” study that included 1,520 patients from April 2020 and February 2021 and showed a total mortality of 3.8% and critical illness in 20% of patients (21). The factors associated with critical illness were low-income or lower-middle-income countries, upper-middle-income countries, teenagers (15–18 years of age), lymphopenia (≤300 cells/mm^3^) and severe neutropenia (≤500 cells/mm^3^). A study in Peru (2020) reported severe morbidity and mortality in patients with new cancer diagnosed during the COVID-19 admission and without previous cancer therapy (22).

In this study, we sought to analyze the risk factors for mortality in children with cancer from multiple Latin American countries during the second year of the pandemic. We hypothesized that patients with new cancer diagnoses had higher mortality than patients with established diagnoses and ongoing cancer therapy.

## Methods

### Study Design and Settings

We performed a multicenter prospective cohort study including data from 12 hospitals in 6 Latin American countries (Argentina, Bolivia, Colombia, Ecuador, Honduras, and Peru). Centers included are listed in the Supplementary Table 1. Ethical approval was obtained in each participating center. The data collection occurred from April 2021 to November 2021. Patients younger than 14 years of age who had a new or established oncologic diagnosis and a diagnosis of COVID-19 or multisystemic inflammatory syndrome (MIS-C) were included. We included data on patients from inpatient and outpatient settings. A descriptive analysis was done for all the patients included. The cohort analysis included data only from the inpatient setting. Study data were collected and managed using REDCap electronic data capture tools hosted at “Fundación Universitaria de Ciencias de la Salud, Bogota Colombia.” (23, 24). The CRF utilized in Spanish is available in the Supplementary Appendix A.

### Patient Groups and Outcome

The primary exposure was diagnosis stage and treatment status. The first group, “new-diagnosis” was defined as patients with no previous diagnosis of cancer, therapy-naïve, and that were diagnosed with cancer during the COVID-19 encounter. The second group, “established diagnosis,” were patients with a known diagnosis of cancer who were on therapy, had not completed initial therapy, and had not achieved remission. A third diagnosis group were patients in “relapse,” defined as patients that had a previous diagnosis of cancer, had a cancer-free period following treatment, and had relapsed active disease on cancer-directed therapy at the time of COVID diagnosis. The diagnosis of relapsed was determined by each treatment group. Another group described in this analysis includes patients in palliative care. This group overlapped with “established diagnosis” and “relapse” groups, and it included patients that were not going to receive further curative cancer treatments.

Clinical data collected for these patients included type of cancer (hematologic vs. solid tumors), the specific cancer diagnosis, and the TNM classification. The data collected about cancer-directed therapy included the dates since last chemotherapy and the diagnosis of COVID-19, if chemotherapy was received within the last 30 days of COVID-19 diagnosis, and if modifications or canceling to chemotherapy were done. Phases of chemotherapy were described as induction, consolidation, and maintenance. Radiotherapy, biologic therapy, and bone marrow transplant data were included. We grouped rituximab, blinatumomab, dasatinib and imatinib as biologic therapies. The primary outcome measured was mortality during the hospital admission. All the data collected is presented in survivors and non-survivors. A descriptive cause of mortality was described for the non-survivors group.

### Variables

The demographic and epidemiological data included the country of origin. The age of patients was divided into three groups: infant and toddlers, including younger or equal than 5 years of age, school age, including children 6–11 years of age and adolescents aged 12–14 years. Comorbidities were categorized as respiratory, cardiovascular, neurological, genetic, and immunological. Information regarding the COVID-19 diagnosis was also collected, including the type of diagnostic test and symptom duration. The clinical presentation of COVID-19 was included for symptomatic patients. The severity of COVID-19 was classified as defined by the World Health Organization as mild, moderate, severe, and critical (25). We also included the patients who met the criteria for multisystem inflammatory syndrome in children (MIS-C) as defined by the CDC (26). The symptoms at presentation were divided as general symptoms, respiratory, cardiovascular, neurological, gastrointestinal, renal, and dermatological. The occurrence of coinfection was also included, defined as a bacterial or fungal infection with a positive culture, or viral detection with serology, antigen, or PCR (polymerase chain rection). The therapy including respiratory support at admission, the highest respiratory support utilized, steroids, vasoactive medications (measured with vaso-inotropic score), intravenous immunoglobulin, antibiotics, anticoagulation, and surgery were also reported (27). We reported the maximum support required and details about the critical presentation included the presence of shock, hemophagocytic syndrome, and organ failure divided by systems. Laboratory data, radiological tests and echocardiogram information were also included. Findings in chest X-rays and computerized tomographies are reported by delimiting the lungs in 4 areas or quadrants (right upper lobe, right lower and middle lobes, left upper lobe, and left lower lobe).

### Statistical Analysis

The data for all the patients is presented as a descriptive analysis for all the variables collected. A frequentist analysis was performed for mortality, including the primary exposure (stage of diagnosis) and all the variables described. The data for inpatient setting is presented as survivors vs. non-survivors. Pearson and Fisher exact test were performed for categorical variables. Wilcoxon rank sum test was performed for all the continuous variables. The data of continuous variables is presented in median and interquartile ranges. A multivariate logistic regression was performed for predictors of mortality. The focus of this analysis was to find variables that could be associated to mortality and that are present on admission. The variables included were stage of diagnosis, type of cancer (hematological vs. solid tumor), co-infection, respiratory, cardiovascular, neurological symptoms, age, time between symptoms and admission, and comorbidities. The results are presented as odds ratio and 95% confidence intervals.

## Results

During the study period we included 226 patients with COVID-19 and cancer. Of those, 210 patients were hospitalized and 30 (14%) of those patients died. The enrollment was mainly from Colombia with 95 (41.6%) patients and Peru with 87 (38.5%) patients. The mortality was higher from Peru, with 19 (25%) patients that died vs. 10 (11%) from Colombia, p 0.003. The rest of the countries did not have mortality reported except Honduras that had 1 patient that died. Demographic data and patients baseline characteristics are presented in Table 1. Patients with low weight-for-age (2 standard deviations below in Z score) had higher mortality; other anthropometric measurements were not associated with mortality.

**Table 1:** Demographic and epidemiological characteristics.

	**All patients**	Inpatient^*^(***n*** **=** **210)**		
	**(*n* = 226)**	**Survivors (*n* = 180)**	**Non-survivors (*n* = 30)**	***p*-Value**
	***n*, (% column) or *p50 [p25–p75]***	***n*, (% row) or *p50 [p25–p75]***	***n*, (% row) or *p50 [p25–p75]***	
**Country**				
Colombia	94 (41.6%)	81(89.0%)	10 (11.0%)	0.003^**^
Peru	87 (38.5%)	57(75.0%)	19(25.0%)	
Ecuador	26 (11.5%)	24(100.0%)	0 (0.0%)	
Argentina	12 (5.3%)	12(100.0%)	0 (0.0%)	
Bolivia	5 (2.2%)	5(100.0%)	0 (0.0%)	
Honduras	2 (0.9%)	1(50%)	1 (50%)	
**Sex**				
Male	116 (51.3%)	92 (86.0%)	15 (14.0%)	0.910
Female	110 (48.7%)	88 (85.4%)	15 (14.6%)	
**Age (years)**	*7.8 (4.0*–*11.1)*	*7.8 (4.0*–*11.3)*	*7.9 (3.8*–*10.9)*	0.884
0–5	90 (39.8%)	71 (85.5%)	12 (14.5%)	0.819
6–11	88 (38.9%)	69 (84.1%)	13 (15.9%)	
12–14	48 (21.2%)	40 (88.9%)	5 (11.1%)	
**Weight, kg**	*25.5 [16.0*–*38.8]*	*24.0 [15.6*–*37.6]*	*27.2 [17.0*–*39.0]*	0.665
**Height, cm**	*123 [102*–*148]*	*122 [102*–*150]*	*126 [103*–*146]*	0.953
*Missing data*	85	61	13	
**BMI**	*17.0 [5.2*–*20.6]*	*16.7 [14.9*–*19.5]*	*20.6 [16.2*–*23.0]*	0.067
*Missing data*	85	61	13	
Anthropometry^***^				
Z score weight x age (<5 years)	*−0.11 [−1.04 y 0.61]*	*−0.16[−1.03 y 0.59]*	*−0.02[−2.54 y 0.80]*	0.872
Low weight (<-2 SD)	7 (9.2%)	3 (40.0%)	3 (60.0%)	0.049
Z score height x age	*−0.94 [−1.70 y 0.26]*	*−0.94 [−1.72 y 0.26]*	*−0.70 [−1.46 y 0.32]*	0.757
Delayed growth (<-2SD)	23 (10.2%)	20 (90.9%)	2 (9.1%)	0.622
*Missing data*	85	61	13	
Z score weight x height (<5 years)	*0.21 [−0.69 y 0.91]*	*0.15 [−0.81 y 0.85]*	*0.15 [0.13 y 0.68]*	0.867
Malnourishment (<-2SD)	5 (6.6%)	4 (80.0%)	1 (20.0%)	0.596
*Missing data*	36	24	6	
Z score BMI x age (> 5 years)	*0.26 [−0.78 y 1.41]*	*0.07 [−0.81 y 1.25]*	*1.36 [0.43 y 1.89]*	0.019
Underweight (<-2SD)	10 (6.7%)	10 (100.0%)	0 (0.0%)	0.456
Eutrophic (-2SD a 2SD)	75 (50.0%)	63 (86.3%)	10 (13.7%)	
Overweight (>2-3SD)	8 (5.3%)	6 (100.0%)	0 (0.0%)	
Obesity (>3SD)	7 (4.7%)	5(71.4%)	2(28.6%)	
*Missing data*	50	37	7	
**Non-oncological comorbidities**				
Any	56 (24.9%)	46 (86.8%)	7 (13.2%)	0.795
Respiratory	7 (3.1%)	5 (100.0%)	0 (0.0%)	0.597
Cardiovascular	10 (4.4%)	8 (80.0%)	2 (20.0%)	0.638
Neurologic	16 (7.1%)	15 (93.8%)	1 (6.3%)	0.478
Genetic	9 (4.0%)	7 (87.5%)	1 (12.5%)	1.000
Immunological	1 (0.4%)	1 (100.0%)	0 (0.0%)	1.000
Other	20 (8.9%)	13 (72.2%)	5 (27.8%)	0.087

*Data presented in absolute numbers and percentage or in medians and interquartile percentiles; Statistical tests only in hospital cases, comparison by chi-square tests (Pearson or Fisher according to number of cases) or by Wilcoxon rank sum for non-parametric distribution continuous variables. Comparison group for categorical variables are all patients that did not meet the condition described. BMI, Body Mass Index; SD, standard deviation*;

* *Defined as stay longer than 24 h*;

** *Only included countries with more than 10 patients*;

*** *Based on World Health Organization tables and classifications*.

### Oncological Characteristics

We found a statistically significant difference between the stage of cancer diagnosis and mortality. There was a statistically significant difference in mortality between patients with new diagnosis (*n* = 11, 36.7%), established diagnosis (*n* = 1, 3.3%) and relapse (*n* = 18, 60%, *p* < 0.001). Four patients who died were on a palliative care. Most patients had hematological cancers (69%) and they had higher mortality (18%) compared to solid tumors (6%, *p* = 0.032). The list of oncologic diagnoses and analyses regarding oncological characteristics and treatments are presented in Table 2. There were 4 (1.3%) patients from the mortality group vs. 1 (0.6%) of the survivors with biological therapy, *p* 0.002.

**Table 2:** Oncological characteristics including diagnosis and oncological therapies.

	**All patients**	Inpatient^*^(***n*** **=** **210)**		
	**(*n* = 226)**	**Survivors (*n* = 180)**	**Non-survivors (n=30)**	***p*-Value**
	***n*, (% column) or *p50 [p25–p75]***	***n*, (% row) or *p50 [p25–p75]***	***n*, (% row) or *p50 [p25–p75]***	
**Diagnostic and therapeutic stage**				<0.001
New diagnosis	84 (37.2%)	70 (86.4%)	11 (13.6%)	
Established diagnosis	82 (36.3%)	72 (98.6%)	1 (1.4%)	
Relapse	60 (26.6%)	38 (67.9%)	18 (32.1%)	
**Hematologic**	162 (71.7%)	120 (82.2%)	26 (17.8%)	0.032^**^
Lymphoid	130 (57.5%)	94 (81.7%)	21 (18.3%)	
Myeloid	21 (9.3%)	17 (85.0%)	3 (15.0%)	
Hodgkin Lymphoma	5 (2.2%)	5 (100.0%)	0 (0.0%)	
Non-Hodgkin Lymphoma	6 (2.7%)	4 (66.7%)	2 (33.3%)	
**Solid tumors**	64 (30.5%)	60 (93.8%)	4 (6.3%)	0.032^**^
Central nervous system	21 (9.3%)	17 (81.0%)	4 (19.0%)	
Osteosarcoma	7 (3.1%)	7 (100.0%)	0 (0.0%)	
Ewing Sarcoma	5 (2.2%)	5 (100.0%)	0 (0.0%)	
Rhabdomyosarcoma	1 (0.4%)	1 (100.0%)	0 (0.0%)	
Wilms Tumor	10 (4.4%)	10 (100.0%)	0 (0.0%)	
Neuroblastoma	3 (1.3%)	3 (100.0%)	0 (0.0%)	
Retinoblastoma	3 (1.3%)	3 (100.0%)	0 (0.0%)	
Germinal cells	4 (1.8%)	4 (100.0%)	0 (0.0%)	
Other	10 (4.4%)	10 (100.0%)	0 (0.0%)	
TNM Staging^***^	74	68 (91.9%)	6 (8.1%)	
0	3 (4.1%)	3 (100.0%)	0 (0.0%)	0.529
I	10 (13.5%)	9 (90.0%)	1 (10.0%)	
II/III	34 (46.0%)	32 (94.1%)	2 (5.9%)	
IV	17 (23.0%)	14 (82.4%)	3 (17.6%)	
Not reported	10 (13.5%)	10 (100.0%)	0 (0.0%)	
**Chemotherapy**				
Received before COVID-19 diagnosis	126 (55.8%)	98 (86.0%)	16 (14.0%)	0.942
Days between first day of chemotherapy and COVID-19 diagnosis	*28 (12–119)*	*28 (11–132)*	*17 (11–45)*	0.347
Received in the previous 30 days	94 (41.6%)	74 (86.0%)	12 (14.0%)	0.909
Days between last therapy and COVID-19 diagnosis	*9 (3–16)*	*9 (3–16)*	*12 (5–17)*	0.613
Suspended therapy due to COVID-19	77 (34.1%)	55 (80.9%)	13 (19.1%)	0.166
Modified drugs or doses due to COVID-19 diagnosis	7 (3.1%)	6 (85.7%)	1 (14.3%)	1.000
**Chemotherapy phase**				
Induction	41 (18.1%)	32 (82.1%)	7 (17.9%)	0.699
Consolidation	35 (15.5%)	28 (84.8%)	5 (15.2%)	
Maintenance	45 (19.9%)	37 (90.2%)	4 (9.8%)	
Unknown	5 (2.2%)	3 (75.0%)	1 (25.0%)	
*Not applicable*	100 (44.2%)	80 (86.0%)	13 (14.0%)	
**Radiotherapy (30 days)**	8 (3.5%)	4 (80.0%)	1 (20.0%)	0.541
Biological therapy^****^(30 days)	5 (2.2%)	1 (20.0%)	4 (80.0%)	0.002
**Bone marrow transplant**	15 (6.6%)	11 (73.3%)	4 (26.7%)	0.239
**Severe neutropenia (<0.5)**	83 (36.7%)	63 (78.0%)	18(22.0%)	0.009

*Data presented in absolute numbers and percentages, or in medians and interquartile percentiles; Statistical tests only in hospital cases, comparison by chi-square tests (Pearson or Fisher according to number of cases) or by Wilcoxon rank sum for non-parametric distribution continuous variables. Comparison group for categorical variables are all patients that did not meet the condition described*.

* *Defined as stay longer than 24 h*;

** *test statistics comparing hematologic and solid tumors*;

*** *TNM: cancer staging system. Proportions over the total number of cancers with the possibility of staging by TNM*;

**** *Rituximab (2), Blinatumomab (1), Dasatinib (1), Imatinib (1)*.

### COVID-19 Characteristics

Table 3 describes details of COVID-19 diagnosis and presentation. Mortality was higher in patients with critical presentation, accounting for 20 (66.7%) of deaths, compared to other COVID-19 severities, *p* 0.001. Seventeen patients that met MIS-C criteria, which was also associated with mortality, 7 (41%) of them died, p 0.001. Respiratory symptoms were more frequent in the non-survivors, specifically respiratory distress, and hypoxemia. From the cardiovascular system, hypotension and slow capillary refill time were associated with higher mortality. Headaches and altered mental status accounted for neurological symptoms associated with mortality. Renal dysfunction symptoms (decreased urine output and edema) were associated with mortality. Finally, patients with coinfections had higher mortality rates. Half of the patients from the mortality group had a bacterial or viral coinfection, and from the mortality group 12 (40%) had a positive bacterial culture. In the survivors, 36 (20%) of the patients had a coinfection and 26 (14.4%) having a positive bacterial or fungal culture, *p* 0.001. The details about the infections are described in the Supplementary Table 2.

**Table 3:** COVID-19 characteristics, diagnostic tests, and symptoms presentation.

	**All patients (*n* = 226)**	Inpatient^*^(***n*** **=** **210)**		
		**Survivors (*n* = 180)**	**Non-survivors (*n* = 30)**	***P***
	***n*, (% column) or *p50 [p25–p75]***	***n*, (% row) or *p50 [p25–p75]***	***n*, (% row) or *p50 [p25–p75]***	
**Asymptomatic**	49 (21.7%)	44 (97.8%)	1 (2.2%)	0.012
**Symptomatic**	177 (78.3%)	139 (82.7%)	29 (17.3%)	
Mild	71 (31.4%)	66 (98.5%)	1 (1.5%)	<0.001
Moderate	25 (11.1%)	20 (87.0%)	3 (13.0%)	
Severe	19 (8.4%)	14 (77.8%)	4 (22.2%)	
Critical	31 (13.7%)	11 (35.5%)	20 (64.5%)	
No classifiable	31 (13.7%)	28 (96.6%)	1 (3.4%)	
**MIS-C**	17 (7.5%)	10 (58.8%)	7 (41.2%)	0.001
Days between symptoms and test^**^	*2 (1–5)*	*2 (1–5)*	*3 (1–9)*	0.083
Days between symptoms and medical attention^**^	*2 (3–9)*	*3 (1–8)*	*7 (2–12)*	0.358
**Positive test on admission**				
RT-PCR	152 (67.3%)	126 (88.1%)	17 (11.9%)	
Antigen	37 (16.4%)	27 (79.4%)	7 (20.6%)	
Antibody	37 (16.4%)	27 (81.8%)	6 (18.2%)	
**Secondary test (>** **10 days)**	17 (7.5%)			
RT-PCR	10 (4.4%)	10 (100.0%)	0 (0.0%)	
Antigen	5 (2.2%)	5 (100.0%)	0 (0.0%)	
Antibody (IgM)	4 (1.8%)	4 (100.0%)	0 (0.0%)	
General symptoms^**^	138 (78.0%)	112 (85.5%)	19 (14.5%)	0.075
Fever	127 (71.8%)	102 (85.0%)	18 (15.0%)	0.220
Asthenia	71 (40.1%)	56 (82.4%)	12 (17.6%)	0.913
Myalgias	34 (19.2%)	30 (88.2%)	4 (11.8%)	0.342
Anosmia/ageusia	9 (5.1%)	8 (100.0%)	0 (0.0%)	0.353
Hyporexia	46 (26.0%)	37 (84.1%)	7 (15.9%)	0.782
Respiratory symptoms^**^	108 (61.0%)	78 (75.0%)	26 (25.0%)	0.001
Cough	93 (52.5%)	69 (77.5%)	20 (22.5%)	0.058
Respiratory distress	51 (28.8%)	30 (60.0%)	20 (40.0%)	<0.001
Hypoxemia	43 (24.3%)	23 (54.8%)	19 (45.2%)	<0.001
Rhinorrhea	41 (23.2%)	35 (85.4%)	6 (14.6%)	0.609
Wheezing	11 (6.2%)	10 (90.9%)	1 (9.1%)	0.692
Stridor	2 (1.1%)	1 (50.0%)	1 (50.0%)	0.316
Cardiovascular symptoms^**^	17 (9.6%)	7 (41.2%)	10 (58.8%)	<0.001
Palpitations	2 (1.1%)	1 (50.0%)	1 (50.0%)	0.316
Hypotension	16 (9.0%)	7 (43.8%)	9 (56.3%)	<0.001
Delayed capillary refill	13 (7.3%)	6 (46.2%)	7 (53.8%)	<0.001
Neurological symptoms^**^	25 (14.1%)	14 (58.3%)	10 (41.7%)	0.001
Headache	16 (9.0%)	9 (60.0%)	6 (40.0%)	0.015
Altered menta status	10 (5.7%)	3 (33.3%)	6 (66.7%)	<0.001
Seizures	6 (3.4%)	3 (50.0%)	3 (50.0%)	0.065
Vertigo	2 (1.1%)	1 (50.0%)	1 (50.0%)	0.316
Paralysis	2 (1.1%)	2 (100.0%)	0 (0.0%)	1.000
Gastrointestinal symptoms^**^	51 (28.8%)	35 (76.1%)	11 (23.9%)	0.254
Vomiting	22 (12.4%)	14 (66.7%)	7 (33.3%)	0.037
Diarrhea	36 (20.3%)	30 (88.2%)	4 (11.8%)	0.450
Abdominal pain	30 (17.0%)	23 (76.7%)	7 (23.3%)	0.332
Jaundice	3 (1.7%)	2 (66.7%)	1 (33.3%)	0.436
Bleeding	3 (1.7%)	1 (33.3%)	2 (66.7%)	0.077
Abdominal distention	9 (5.1%)	5 (55.6%)	4 (44.4%)	0.049
Renal symptoms^**^	12 (6.8%)	6 (50.0%)	6 (50.0%)	0.002
Oligo-anuria	12 (6.8%)	6 (50.0%)	6 (50.0%)	0.002
Edema	7 (4.0%)	2 (28.6%)	5 (71.4%)	0.002
Proteinuria/hematuria	3 (1.7%)	1 (33.3%)	2 (66.7%)	0.077
Dermatological symptoms^**^	8 (4.5%)	8 (100.0%)	0 (0.0%)	0.353
Acral lesions	1 (0.6%)	1 (100.0%)	0 (0.0%)	1.000
Urticaria	1 (0.6%)	1 (100.0%)	0 (0.0%)	1.000
Rash	6 (3.4%)	6 (100.0%)	0 (0.0%)	0.591
Other symptoms^**^	6 (3.4%)	6 (100.0%)	0 (0.0%)	0.591
**Additional microorganisms identified**	53 (23.5%)	36 (70.6%)	15 (29.4%)	<0.001
Viral antigen	3 (1.3%)	3 (100.0%)	0 (0.0%)	1.000
Viral PCR	5 (2.2%)	3 (60.0%)	2 (40.0%)	0.150
Viral antibody	9 (4.0%)	8 (88.9%)	1 (11.1%)	1.000
Bacterial cultures	40 (17.7%)	26 (68.4%)	12 (31.6%)	0.001

*Data presented in absolute numbers and percentage; Statistical tests only in hospital cases, comparison by chi-square tests (Pearson or Fisher according to number of cases); MISC, Multisystem inflammatory syndrome associated with COVID-19. Comparison group for categorical variables are all patients that did not meet the condition described*.

* *Defined as stay longer than 24 h*;

** *Proportions on symptomatic patients only*.

We also collected laboratory information at admission. Mortality was associated with acidosis, hypercapnia, hypoxemia, PaO2/FiO2 ratio of < 100, lactic acidosis, renal failure, anemia, severe thrombocytopenia, severe neutropenia, severe lymphopenia, liver failure and hyperferritinemia. These results are presented in the Supplementary Table 3. Regarding radiological and imaging studies, 75% of the patients had a chest X-ray and 39% a chest computerized tomography (CT). There was no difference in mortality associated to the number of quadrants with pulmonary infiltrates. However, patients with pleural effusion, 16 (55.3%) of non- survivors vs. 12 (6.7% of survivors, or pneumothorax, 9 (30%) of survivors vs. 7 (3.8%) had higher mortality, *p* 0.001. The details of this studies are presented in the Supplementary Table 4.

### Description of Patients With Critical Disease and Maximum Therapy Utilized

Shock was present more frequently in non-survivors, 25 (83.3%) compared to survivors 26 (14.4%). Multiorgan failure was also more frequent in the mortality group. On admission, most survivors did not require respiratory support, 121 (67.2%) compared to 22 (73.3%) of patients that died. The highest respiratory support during the admission also was different: 20 (66.7%) of the patients that died require invasive mechanical ventilation compared to 107 (59.4%) of the survivors that remained in room air. Vasoactive drugs were used in 23 (76.7%) of the patients that died vs. 23 (12.8%) of the survivors. The details are provided in Table 4.

**Table 4:** Critical presentation, and support therapies.

	**All (*n* = 226)**	Inpatient^*^(***n*** **=** **210)**	
		**Survivors (*n* = 180)**	**Non-survivors (*n* = 30)**
	***n*, (% column) or *p50 [p25–p75]***	***n*, (% row) or *p50 [p25–p75]***	***n*, (% row) or *p50 [p25–p75]***
**Shock**	51 (22.6%)	26 (51.0%)	25 (49.0%)
Septic	44 (19.5%)	20 (46.5%)	23 (53.5%)
Cardiogenic	14 (6.2%)	6 (42.9%)	8 (57.1%)
Vasoplegic	15 (6.6%)	5 (33.3%)	10 (66.7%)
Hypovolemic	5 (2.2%)	3 (60.0%)	2 (40.0%)
**Hemophagocytic syndrome**	3 (1.3%)	1 (33.3%)	2 (66.7%)
**System dysfunction**	62 (27.4%)	36 (58.1%)	26 (41.9%)
Cardiovascular	33 (14.6%)	15 (45.5%)	18 (54.5%)
Hematologic	48 (21.2%)	29 (60.4%)	19 (39.6%)
Liver	11 (4.9%)	5 (45.5%)	6 (54.5%)
Neurologic	13 (5.8%)	2 (15.4%)	11 (84.6%)
Renal	24 (10.6%)	10 (41.7%)	14 (58.3%)
Respiratory	41 (18.1%)	17 (41.5%)	24 (58.5%)
**Initial respiratory support**			
IMV	6 (2.7%)	2 (33.3%)	4 (66.7%)
HFNC	3 (1.3%)	3 (100.0%)	0 (0.0%)
High flow	20 (8.9%)	11 (55.0%)	9 (45.0%)
Low flow	53 (23.5%)	43 (82.7%)	9 (17.3%)
Room air	144 (63.7%)	121 (93.8%)	8 (6.2%)
**Maximal respiratory support**			
IMV	33 (14.6%)	13 (39.4%)	20 (60.6%)
HFNC/ CPAP/ NIV	7 (3.1%)	5 (71.4%)	2 (28.6%)
High flow	14 (6.2%)	11 (78.6%)	3 (21.4%)
Low flow	48 (21.2%)	44 (93.6%)	3 (6.4%)
Room air	124 (54.9%)	107 (98.2%)	2 (1.8%)
**Steroids**	65 (28.8%)	46 (73.0%)	17 (27.0%)
Dexamethasone	49 (21.7%)	34 (70.8%)	14 (29.2%)
Prednisone	5 (2.2%)	5 (100.0%)	0 (0.0%)
Methylprednisolone	13 (5.8%)	9 (75.0%)	3 (25.0%)
Pulse	2 (0.9%)	1 (50.0%)	1 (50.0%)
**Vasoactive**	46 (20.4%)	23 (50.0%)	23 (50.0%)
VIS	40 (12–60)	30 (42.9%)	40 (57.1%)
**Immunoglobulin**	23 (10.2%)	18 (81.8%)	4 (18.2%)
**Antibiotic**	155 (68.6%)	122 (80.8%)	29 (19.2%)
**Anticoagulation**			
None	189 (83.6%)	151 (86.3%)	24 (13.7%)
Therapeutical	16 (7.1%)	11 (78.6%)	3 (21.4%)
Prophylactical	21 (9.3%)	18 (85.7%)	3 (14.3%)
**Surgery**	39 (17.3%)	35 (89.7%)	4 (10.3%)

*Data presented in absolute numbers and percentage or in medians and interquartile percentiles; Statistical tests only in hospital cases, comparison by chi-square tests (Pearson or Fisher according to number of cases) or by Wilcoxon rank sum for non-parametric distribution continuous variables; IMV, invasive mechanical ventilation; HFNC, high-flow nasal cannula; CPAP, continuous positive pressure; NIV, non-invasive ventilation; VIS, vasoactive inotropic score; ICU, intensive care unit*;

* *Defined as a stay of more than 24 h*.

### Cause of Death

The causes of death were diverse; 6 (20%) of the patients died from a cancer or cancer treatment-related complications. Two of those had febrile neutropenia, one had complications of tumor lysis syndrome, three had advanced stages of their cancer. One of the patients from this group was in the palliative group. 21 (70%) patients died from septic shock, respiratory failure or multiorgan failure. In all those cases the cause of mortality was attributed to the COVID-19 infection. Three of those patients were in the palliative group. 3 (10%) patients died from hemorrhagic complications, two intracranial bleeds and one pulmonary hemorrhage. These patients represent a combination of complications from COVID-19 infection and high-risk cancer presentations.

### Logistic Regression for Mortality

**Figure 1:**
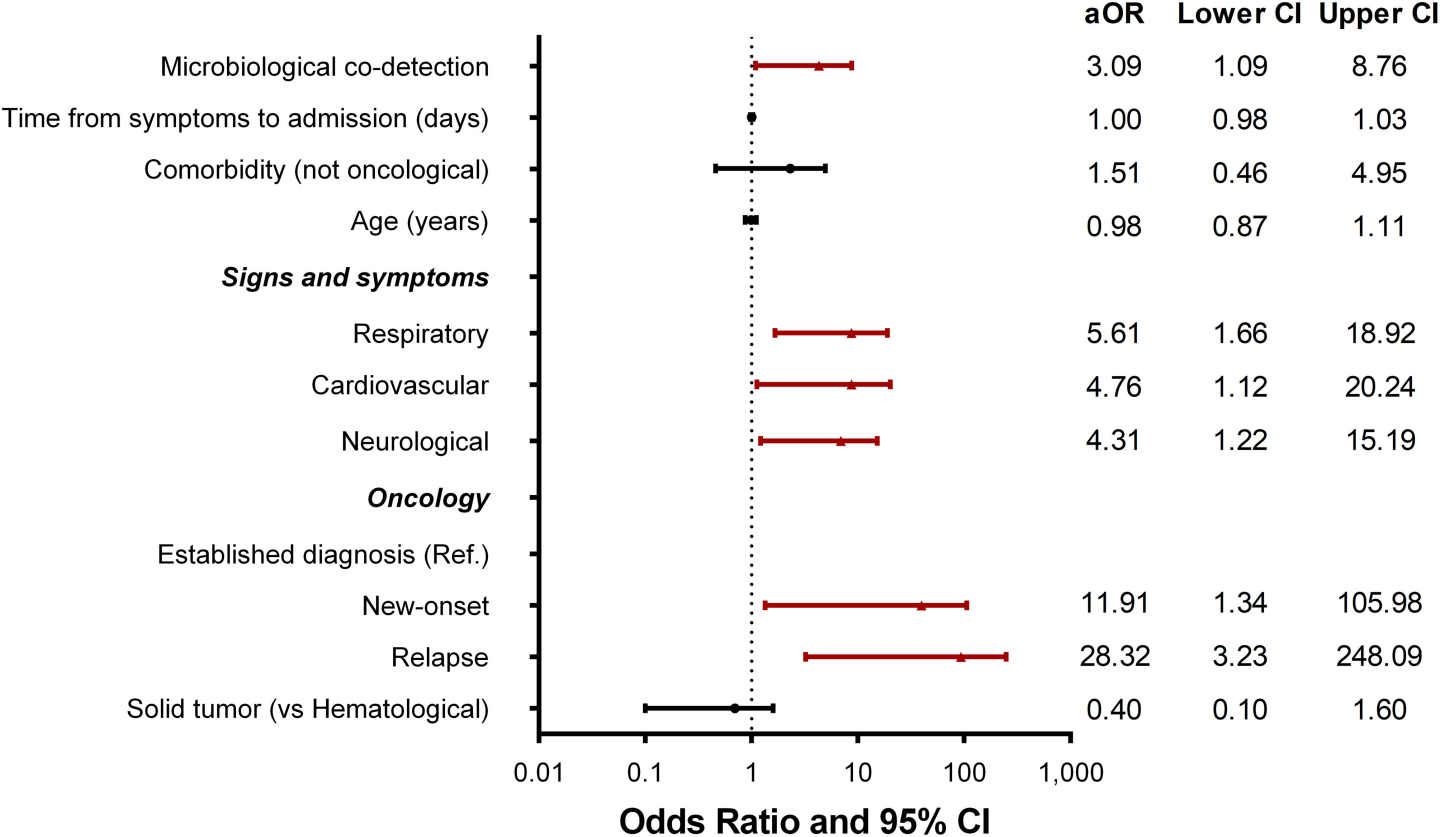
Forest plot for odds–ratio and 95% confidence intervals from multivariate logistic regression model.

A multivariate logistic regression model was performed including variables significant for mortality at presentation from the primary analysis. This model showed factors associated with mortality. Patients with a new cancer diagnosis had an OR 11.5 95%CI (1.31–102) and relapse OR 25 95%CI (2.9–214) (established cancer is the reference). Other factors with higher odds of mortality include concomitant bacterial infection, respiratory symptoms, and cardiovascular symptoms at admission. The details are presented in Figure 1.

## Discussion

The primary objective of our study was to determine if the stage of oncological diagnosis was associated to higher mortality in pediatric patients with COVID-19. Our study found that patients with relapsed oncologic disease and with those newly diagnosed cancer had higher mortality. Upon analyzing the clinical presentation of these patients, we identified that respiratory, cardiovascular, and neurological symptoms were associated with increased mortality.

One of the strengths of our study is to present data for this specific population in Latin American countries and analyze factors of mortality specific for our region during 2021. The global characteristics and outcomes of SARS-CoV-2 infection in children and adolescents with cancer (GRCCC) reported that the all-cause mortality in that study was 6.3%, ranging from 11% in low-middle income countries to 6% in upper-middle income countries (21). The mortality attributed to COVID-19 in this population reported by multiple studies from different countries in Latin America ranged from 6 to 12.3% (28–31). A study by Shahid et al. performed in Pakistan, included 161 cancer patients with and without COVID-19 and compared their outcomes, finding a mortality of 6.8% in COVID-19 patients vs. 2.7% non-COVID-19 patients (32). Meena et al. published a systematic review that included 226 pediatric patients with cancer and COVID-19, showing that 10% of these patients had a critical presentation, requiring critical care admission, and a mortality rate of 4.9% (33). The mortality measured by case fatality rate in pediatric patients with COVID-19 and without cancer reported in different studies from Latin-American varies from 8 to 20% (34–36). However, the mortality and the need for critical care in our study is higher than the findings of other studies looking at patient with cancer and COVID-19. One factor that could explain this difference is that their data is presented for countries from different regions that had different access to medical care during the pandemic. We have a heterogeneous distribution of patients amongst the countries included. However, analyzing mortality factor for each center wouldn't be possible with the number of patients included. Another factor that may explain this difference in mortality is the effect of different variants. Although we did not gather data on variants in our study, our study was conducted during the peak of the Delta variant in our region (37). Our primary objective was to ascertain the mortality associated with different stages of diagnosis including patients that presented with severe forms of COVID-19 at the time of oncological diagnosis. In our study, patients with a new diagnosis of cancer who were therapy naïve also had higher mortality than patients with a prior diagnosis of cancer and ongoing chemotherapy. Further studies would be necessary to determine the immunological impairment of patients with undiagnosed cancer and the possibility of developing a critical form of COVID-19.

In our study, hematological cancer had higher mortality. However, in the regression analysis that was not demonstrated. In the GRCCC study, patients with malignant hematological neoplasms contributed the highest proportion of severe or critical illness (21). In patients with acute lymphoblastic leukemia or acute lymphoblastic lymphoma, severe or critical illness was more common in those who received induction therapy, relapsed or refractory therapy, and the maintenance or continuation phase of therapy. Like GRCCC findings, our study patients with severe neutropenia or severe lymphopenia had higher mortality. We found that concomitant bacterial infection was also associated with mortality. Concomitant bacterial infections in patients with COVID-19 have not been a concern for higher mortality (38). However, bacterial infections in febrile neutropenic patients are a known contributing factor of critical disease and mortality (39, 40). More detailed data regarding the specific chemotherapies utilized for each patient, their microbiological history and timing of the cultures would be necessary to determine if concomitant infections in oncological patients and COVID-19 have a higher risk of mortality than oncological patients without SARS-CoV-2 infection.

The clinical presentation and laboratory profile findings matched the characteristics of patients with severe and critical COVID-19 presentation. There are specific signs and symptoms associated with severe disease described in our study that should trigger the concern for critical disease and mortality. In the regression analysis, the patients that presented with respiratory cardiovascular and neurological symptoms were associated with increased mortality. As described for the general population respiratory symptoms are the most common finding with COVID-19. In our study population respiratory involvement presented as respiratory distress and hypoxemia; cardiovascular involvement with hypotension and prolonged capillary refill and neurological involvement presented with headache and altered mental status and all the above findings were associated with mortality. The symptoms included in the severity criteria for COVID-19 by the World Health Organization include hypoxemia and respiratory distress (25). The frequency of neurological and cardiovascular symptoms in our cohort is low. However, those symptoms found in our study have been associated to severe COVID-19 in other studies (41, 42). More information regarding the etiology of neurological symptoms (COVID-19 vs. oncological disease) is necessary to determine the predictability for severe disease in this population. Cardiovascular symptoms seen in our study are associated to shock which will be a universal severity criterion. Severe neutropenia in our cohort was associated with higher rate of mortality. Studies report mortality for patients with fever and neutropenia as high as 75% (43). Specific comparative studies of patients with and without covid and neutropenia are necessary. The association of bacterial infections and mortality seen in our cohort has been reported in other studies as risk factor for mortality. The patients from our cohort that presented with MIS-C were also associated to higher mortality. Diagnostic criteria of MIS-C can overlap with inflammatory symptoms of cancer and have a strong association with cardiovascular shock (44). Identification of patients at risk for severe disease, critical disease, and mortality in our population is not different than the clinical presentations described by other studies. Early identification of these patients and instructions to the parents with cancer regarding alarm signs can help starting appropriate medical interventions to minimize complications. Further studies specific for MIS-C and COVID-19 are necessary to determine specific and differential symptoms between both with pediatric patients with undiagnosed cancer. A major concern of patients with cancer and COVID-19 are the side effects of therapies and the impact of modification of cancer-directed therapy during the pandemic (17). Authors of a Polish study concluded that the SARS-CoV-2 infection did not increase mortality immediately in patients with cancer, but it was associated to higher chemotherapy rates discontinuation (45). Similar results were obtained by Parambil et al. and the authors concluded that chemotherapy shouldn't be interrupted upon obtaining a positive test identifying SARS-CoV-2 (46). A pending question is if chemotherapy can be continued in patients with severe disease and improve recommendations of treatment for patients with cancer and COVID-19 (47).

Our study has several limitations. We did not have a control group of patients with COVID-19 without cancer during the same period. This limited our ability to analyze if the symptoms, laboratory, and radiographic findings identified in our study are specific to patients with cancer in addition to the variations in each center included. The number of patients included is small and identifying factors associated to mortality in such complex population is difficult. Efforts to minimize selection bias and including all the patients with COVID-19 and cancer in each center were directed by the study investigators in their own centers. The variability observed between countries in enrollment and mortality are probably explained by the capture of all patients with COVID-19 and cancer.

New questions arise from our cohort study. More detailed information regarding the immunogenicity of undiagnosed patients with cancer and the possibility of severe disease with COVID-19 can help multidisciplinary decisions regarding the timing and intensity of treatments. More information regarding patients with relapse therapies and risks for severe disease is needed. Larger studies to analyze differences of chemotherapy drugs and adjusting by characteristics of each center would be helpful. Finally, new studies including the impact of vaccines in this population will be necessary.

## Conclusion

Our study shows that pediatric patients with newly diagnosed cancer and patients with relapsed disease have higher odds of mortality in the setting of COVID-19 infection. Pediatric patients with cancer and COVID-19 in Latin America have risk factors associated with mortality, including a clinical presentation with respiratory, neurological, and cardiovascular symptoms. This information would help develop an early identification of patients with cancer and COVID-19 with higher risk of mortality.

## Data Availability Statement

The original contributions presented in the study are included in the article/Supplementary Material, further inquiries can be directed to the corresponding author.

## Ethics Statement

The studies involving human participants were reviewed and approved by Protocolo aprobado IRB Universidad Austral Comité Institucional de Evaluación depende del CEC central Buenos Aires - Argentina P21-035. Written informed consent from the participants' legal guardian/next of kin was not required to participate in this study in accordance with the national legislation and the institutional requirements.

## Author Contributions

JD-R, PV-H, LV-P, and AC-M had full access to all of the data in the study, takes responsibility for the integrity of the data and the accuracy of the data analysis, and drafting of the manuscript. All the authors contributed equally. JD-R, PV-H, AC-M, LM-R, VA-A, and SG-D: concept and design. JD-R, PV-H, RP-M, AM-Q, LM-R, AD-D, ST, AC-D, LC-B, VA-A, AC, AA, SM, GA-G, NR-S, AM-Z, MT-P, LV-P, RL-P, MP-A, MT-H, JM-M, CP, JL, SG-D, and AC-M: acquisition, analysis, and interpretation of data. JD-R, PV-H, AC-M, LV-P, AC, LM-R, VA-A, SG-D, AA, and SM: critical revision of the manuscript for important intellectual content. PV-H, AC-M, and JD-R: statistical analysis. JD-R: obtained funding. All authors are accountable for all aspects of the work in ensuring that questions related to the accuracy or integrity of any part of the work are appropriately investigated and resolved, approved the final version for submission, and contributed equally in this manuscript.

## Conflict of Interest

The authors declare that the research was conducted in the absence of any commercial or financial relationships that could be construed as a potential conflict of interest.

## Publisher's Note

All claims expressed in this article are solely those of the authors and do not necessarily represent those of their affiliated organizations, or those of the publisher, the editors and the reviewers. Any product that may be evaluated in this article, or claim that may be made by its manufacturer, is not guaranteed or endorsed by the publisher.
