# Supplementary material for: Association of Cancer Diagnosis and Therapeutic Stage With Mortality in Pediatric Patients With COVID-19, Prospective Multicenter Cohort Study From Latin America
Source: Front Pediatr. 2022 May 3;10:885633. doi: 10.3389/fped.2022.885633 (PMC9110860; doi:10.3389/fped.2022.885633)
Supplement: Supplementary file 1 [file Data_Sheet_1.pdf]

Table S1, Center by country	
Argentina	Hospital Universitario Austral Pilar, Buenos Aires
Bolivia	Hospital del Niño Doctor Ovidio Aliaga Uria, La Paz
Colombia	Universidad Nacional de Colombia and Sociedad de Cirugía Hospital de San José, FUCS, Bogotá
	Hospital General de Medellín, Medellín.
	Fundación Hospital Pediátrico La Misericordia, Bogotá
	Fundación Valle del Lili, Cali
	Hospital Pablo Tobon Uribe
	Clínica Blas de Lezo, Cartagena
Ecuador	Instituto del Cáncer SOLCA Cuenca, Universidad de Cuenca – Azuay
Honduras	Hospital del Norte, IHSS, San Pedro Sula
Peru	Hospital Edgardo Rebagliati Martins, Lima
	Hospital Nacional Carlos Alberto Seguin Escobedo Essalud, Arequipa

Table S2: detailed description of concomitant infections (CMV, Cytomegalovirus; CSF, Cerebrospinal fluid)			
**Cultures**		38	18.10%
Blood	*Acinetobacter baumannii*	3	1.4%
	Cryptococcus neoformans	1	0.5%
	Escherichia coli	6	2.9%
	Streptococcus viridians	2	1.0%
	Staphylococcus aureus	5	2.4%
	Staphylococcus - coagulase negative	3	1.4%
	Enterobacter cloacae	1	0.5%
	Bacillus cereus	1	0.5%
	Enterococcus faecium	1	0.5%
	Klebsiella pneumoniae	2	1.0%
	Pseudomonas aeruginosa	4	1.9%
	Streptococcus pneumoniae	1	0.5%
Urine	*Serratia marcescens*	1	0.5%
	Escherichia coli	2	1.0%
	Klebsiella pneumoniae	1	0.5%
	Enterobacter cloacae	1	0.5%
Trachea/ respiratory	*Streptococcus viridians*	1	0.5%
	Acinetobacter baumannii	1	0.5%
	Streptococcus pneumoniae	1	0.5%
	Candida albicans	1	0.5%
Pleural	*Klebsiella pneumoniae*	1	0.5%
	Escherichia coli	1	0.5%
CSF	*Pseudomonas aeruginosa*	1	0.5%
	Enterococcus faecalis	1	0.5%
	Staphylococcus - coagulase negative	1	0.5%
	Candida albicans	1	0.5%
Tissue	*Aspergillus*	1	0.5%
Eye drainage	*Pseudomonas spp.*	1	0.5%
Stool culture	*Salmonella typhi*	1	0.5%
**Molecular**		5	2.4%
	Clostridiodes difficile	2	1.0%
	Rhinovirus/enterovirus	1	0.5%
	Citomegalovirus, viral load	1	0.5%
	Epstein Barr, viral load	1	0.5%
**Antigen**		3	1.4%
	Rhinovirus	2	1.0%
	Adenovirus	1	0.5%
**Antibodies**		9	4.3%
	Citomegalovirus	5	2.4%
	Epstein Barr	6	2.9%
	Dengue	1	0.5%

Table S3: laboratory values and oxygenation calculations				
	All patients (n=226)	Inpatient* (n=210)		
		Survivors (n=180)	Non-survivors (n=30)	p-value
**Acid-base**				
pH	7.34 (7.28-7.40)	7.35 (7.31-7.41)	7.25 (7.10-7.36)	0.002
PCO_2	44 (35-48)	43 (32-47)	46 (38-63)	0.038
HCO_3	21 (18-23)	21 (19-23)	19 (14-24)	0.162
missing value	*144 (63.7%)*	*121 (67.2%)*	*8 (26.7%)*	
**Oxygenation (arterial)**				
PaO_2	71 (56-86)	75 (57-89)	64 (50-73)	0.095
FiO_2	0.45 (0.28-1.00)	0.38 (0.28-0.65)	1.00 (1.00-1.00)	<0.001
PaO_2/FiO_2	195 (71-286)	221 (90-321)	65 (50-178)	0.001
PaO_2/FiO_2 <100	21 (9.3%)	10 (5.6%)	11 (36.7%)	<0.001
missing value	*173 (76.5%)*	*142 (78.9%)*	*15 (50.0%)*	
**Oxygenation (peripheric)**				
Oximetry (SpO_2)	94 (88-98)	94(90-98)	84(70-88)	<0.001
SpO_2 < 90%	66 (29.2%)	42(23.3%)	23 (76.7%)	<0.001
FiO_2	0.21 (0.21-0.35)	0.21 (0.21-0.28)	1.00 (0.24-1.00)	<0.001
SpO_2/FiO_2	448 (250-467)	448 (314-467)	88 (70-292)	<0.001
*FiO_2 not available**	*26 (11.5%)*	*18 (10.0%)*	*1 (3.3%)*	
**Hematological**				
Hemoglobin (g/dL)	9.3 (7.6-11.3)	9.4 (7.7-11.4)	7.2 (6.2-9.6)	0.001
missing value	*14 (6.2%)*	*9 (5.0%)*	*1 (3.3%)*	
Leucocytes (10^3/uL)	3.97 (1.25-9.68)	4.10 (1.60-9.86)	1.24 (0.21-9.79)	0.102
Neutropenia severe (<0.5)	83 (36.7%)	63 (35.0%)	18(60.0%)	0.009
Lymphopenia severe (<1.0)	98 (43.4%)	73(40.6%)	21(70.0%)	0.003
missing value	*10 (4.4%)*	*5 (2.8%)*	*1 (3.3%)*	
Platelets (10^3/uL)	106 (32-251)	120 (38-259)	12 (8-186)	0.001
missing value	*10 (4.4%)*	*5 (2.8%)*	*1 (3.3%)*	
**Coagulation**				
Prothrombin Time (PT) (seconds)	13.9 (12.0-17.0)	13.6 (12.0-16.0)	15.3 (12.5-19.1)	0.108
missing value	*82 (36.3%)*	*63 (35.0%)*	*4 (13.3%)*	
International normalized ratio [INR]	1.2 (1.1-1.5)	1.1(1.0-1.4)	1.5 (1.2-1.8)	0.004
missing value	*89 (39.4%)*	*69 (38.3%)*	*5 (16.7%)*	
Partial thromboplastin time activated (PTTa) (seconds)	36.0 (31.5-42.0)	35.3 (30.9-40.3)	39.0 (34.0-52.9)	0.029
missing value	*87 (38.5%)*	*67 (37.2%)*	*5 (16.7%)*	
Fibrinogen (mg/dL)	301 (194-505)	297 (186-467)	471 (248-767)	0.085
missing value	*94 (41.6%)*	*77 (42.8%)*	*8 (26.7%)*	
D-Dimer (mg/dL)	342 (8-1000)	350 (44-992)	671 (4-3032)	0.2456
missing value	*131 (58.0%)*	*111 (61.7%)*	*11 (36.7%)*	
**Renal**				
Blood urea nitrogen [BUN] (mg/dL)	15.0 (9.0-25.3)	13.2 (8.1-21.4)	32.9 (15.0-56.0)	<0.001
missing value	*67 (29.6%)*	*50 (27.8%)*	*8 (26.7%)*	
Creatinine (mg/dL)	0.40 (0.30-0.56)	0.39 (0.30-0.52)	0.58 (0.38-1.00)	0.003
missing value	*24 (10.6%)*	*15 (8.3%)*	*2 (6.7%)*	
**Liver**				
Aspartate aminotransferase (AST) (U/L)	35 (23-61)	31 (22-46)	76 (36-204)	<0.001
missing value	41 (18.1%)	31 (17.2%)	3 (10.0%)	
Alanine transaminase (ALT) (U/L)	34 (19-67)	30 (18-54)	47 (21-134)	0.044
missing value	39 (17.3%)	29 (16.1%)	3 (10.0%)	
**Metabolic**				

Lactate (mmol/L)	2.0 (1.0-3.4)	1.8 (1.0-3.0)	4.0 (2.3-6.6)	<0.001
missing value	*124 (54.9%)*	*106 (58.9%)*	*8 (26.7%)*	
Triglycerides (mg/dl)	152 (102-224)	150 (100-213)	258 (150-522)	0.026
missing value	*163 (72.1%)*	*127 (70.6%)*	*23 (76.7%)*	
Lactate Dehydrogenase [LDH] (U/L)	337 (202-551)	275 (190-460)	757 (457-1886)	<0.001
missing value	*74 (32.7%)*	*61 (33.9%)*	*5 (16.7%)*	
Glucose (mg/dl)	98 (88-110)	96 (87-108)	133 (91-206)	0.001
missing value	*65 (28.8%)*	*54 (30.0%)*	*3 (10.0%)*	
Albumin (g/dl)	3.5 (2.8-4.0)	3.7(3.0-4.2)	2.9 (2.3-3.4)	0.002
missing value	*115 (50.9%)*	*96 (53.3%)*	*9 (30.0%)*	
Sodium (mmol/L)	138 (135-139)	138(135-139)	140 (138-145)	0.002
missing value	*42 (18.6%)*	*32 (17.8%)*	*2 (6.7%)*	
Potassium (mmol/L)	3.9 (3.5-4.1)	3.9 (3.5-4.1)	3.7 (2.1-4.3)	0.403
missing value	*78 (34.5%)*	*57 (31.7%)*	*13 (43.3%)*	
Phosphorus (mg/dL)	4.0 (3.0-5.0)	4.0 (3.4-5.0)	4.0 (1.8-5.0)	0.395
missing value	*125 (55.3%)*	*96 (53.3%)*	*19 (63.3%)*	
Uric acid (mg/dL)	4.0 (3.0-5.3)	4.0(3.0-5.2)	3.8(2.6-5.6)	0.853
missing value	*149 (65.9%)*	*116 (64.4%)*	*18 (60.0%)*	
**Other**				
CRP (mg/dl)	5.0 (1.2-15.9)	4.6(1.1-14.2)	15.9(9.4-30.0)	0.000
missing value	*23 (10.2%)*	*17 (9.4%)*	*1 (3.3%)*	
Ferritine (ng/mL)	700 (296-2363)	547 (222-1856)	3500 (1370-6700)	0.000
missing value	*139 (61.5%)*	*116 (64.4%)*	*13 (43.3%)*	
Troponine I	0.07 (0.02-0.20)	0.15 (0.02-0.20)	0.02 (0.02-0.14)	0.267
missing value	*200 (88.5%)*	*163 (90.6%)*	*21 (70.0%)*	

**Table S4 Radiological findings**				
	All (n=226)	Inpatient* (n=210)		
		Survivors (n=180)	Non-survivors (n=30)	p-value
**Echocardiogram**	65 (28.8%)	52 (28.9%)	13 (44.3%)	0.113
Dilatation coronaries/aneurism	3 (1.3%)	2(1.1%)	1(3.3%)	0.372
Pericarditis	14 (6.2%)	10(5.6%)	4(13.3%)	0.121
Other	10 (4.4%)	5(2.8%)	5(16.7%)	0.001
**Chest X-Ray**				
Normal	168 (74.3%)	136 (75.6%)	29 (96.7%)	0.007
Opacities in 1 quadrant	68 (30.1%)	64 (35.6%)	2 (6.7%)	0.001
Opacities in 2 quadrants	21 (9.3%)	19 (10.6%)	2(6.7%)	0.745
Opacities in 3 quadrants	34 (15.0%)	27(15.0%)	6(20.0%)	0.486
Opacities in 4 quadrants	6 (2.7%)	5(2.8%)	1(3.3%)	1.000
Pleural effusion	28 (12.4%)	12(6.7%)	16(55.3%)	<0.001
Pneumothorax	16 (7.1%)	7(3.8%)	9(30.0%)	<0.001
Other	4 (1.8%)	2 (1.1%)	2 (6.7%)	0.099
**Chest tomography**	17 (7.5%)	14 (7.8%)	3(10.0%)	0.716
Normal	80 (35.4%)	61(33.9%)	18(60.0%)	0.006
Opacities in 1 quadrant	12 (5.3%)	10 (5.6%)	2(6.7%)	0.683
Opacities in 2 quadrants	9 (4.0%)	8(4.4%)	1(3.3%)	1.000
Opacities in 3 quadrants	27 (12.0%)	23(12.8%)	4(13.3%)	1.000
Opacities in 4 quadrants	8 (3.5%)	7(3.9%)	1(3.3%)	1.000
Pleural effusion	16 (7.1%)	7(3.9%)	9(30.0%)	<0.001
Pneumothorax	14 (6.2%)	8(4.4%)	6(20.0%)	0.002
Other	2 (0.9%)	1(0.6%)	1(3.3%)	0.266
**Other imaging studies (abnormal)**	12 (5.3%)	10(5.6%)	1(3.3%)	1.000
Brain	34 (15.0%)	24(13.3%)	10(33.3%)	0.006
Renal	8 (3.5%)	3(1.7%)	5(16.7%)	0.002
Liver	4 (1.8%)	4(2.2%)	0	1.000
Other	12 (5.3%)	10(5.6%)	2(6.7%)	0.683

*Data presented in absolute numbers and percentage; Statistical tests only in hospital cases comparison by chi-square tests (Pearson or Fisher according to number of cases); *Defined as stay greater than 24 hours.*
