# Supplementary material for: Association of Cancer Diagnosis and Therapeutic Stage With Mortality in Pediatric Patients With COVID-19, Prospective Multicenter Cohort Study From Latin America
Source: Front Pediatr. 2022 May 3;10:885633. doi: 10.3389/fped.2022.885633 (PMC9110860; doi:10.3389/fped.2022.885633)
Supplement: Supplementary file 2 [file Data_Sheet_2.pdf]

IDENTIFICADOR	1-1
Fecha de nacimiento	[*DATA REMOVED*]<br>(año-mes-día)
Fecha de atención	[*DATA REMOVED*]<br>(año-mes-día)
Sexo	⊗ Masculino<br>◯ Femenino
Edad (años)	0.8<br>(Ojo edad limite < 15)
Peso (kilogramos)	7.200<br>(permite decimales.)
Talla (centimetros)	77
Indice de masa corporal	12.14
¿Tiene alguna comorbilidad no relacionada con la enfermedad oncológica?	◯ No<br>⊗ Si
¿Qué categoría compromete la comorbilidad?	☐ Prematurez<br>☐ Respiratorias<br>☐ Cardiovasculares<br>☐ Neurológicas<br>☐ Genéticas<br>☐ Inmunológicas<br>☒ Otras<br>(Escoge los sistemas en los cuales se presentaron los síntomas del paciente)
Describir el detalle de las comorbilidades	[*DATA REMOVED*]

ONCOCOVID

GRAVEDAD DE COVID-19 DEFINIDO POR OMS
basado en compromiso pulmonar. (Escoja SOLO una opción)
NO CLASIFICABLE: No presenta síntomas pulmonares que permitan clasificarlo. LEVE: Pacientes sintomáticos respiratorio que se ajustan a la definición de caso de COVID-19 pero no presentan neumonía vírica ni hipoxia. MODERADA: Pacientes con signos clínicos de neumonía (fiebre, tos, disnea, taquipnea) pero sin signos de neumonía grave, en particular SpO2 ≥ 90% con aire ambiente. GRAVE: Pacientes con neumonía grave (fiebre, tos, disnea, taquipnea) más alguno de los siguientes: taquipnea para la edad, disnea grave (quejido, tiraje costal muy intenso) o SpO2 < 90% en aire ambiente, signos generales de alerta (incapacidad de mamar o beber, paciente aletargado, inconsciente o con convulsiones) CRITICA: Paciente presenta alguno de los siguientes síndromes: Síndrome de dificultad respiratoria aguda (SDRA), Sepsis o Choque séptico. CRITERIOS CDC MIS-C
Un individuo menor de 21 años que presenta fiebre* Evidencia de laboratorio de inflamación Evidencia de enfermedad clínicamente grave que requiera hospitalización, con afectación de órganos multisistémicos (> 2) (cardíacos, renales, respiratorios, hematológicos, gastrointestinales, dermatológicos o neurológicos) Sin diagnósticos plausibles alternativos Positivo para infección actual o reciente por SARS-CoV-2 por RT-PCR, serología o prueba de antígeno; o exposición a un caso de COVID-19 sospechado o confirmado dentro de las 4 semanas previas al inicio de los síntomas. * Fiebre> 38.0 ° C durante ≥24 horas, o informe de fiebre subjetiva que dura ≥24 horas**
**** Incluyendo, entre otros, uno o más de los siguientes: proteína C reactiva (PCR) elevada, velocidad de sedimentación globular (VSG), fibrinógeno, procalcitonina, dímero D, ferritina, deshidrogenasa del ácido láctico (LDH), o interleucina 6 (IL-6), neutrófilos elevados, linfocitos reducidos y albúmina baja**

Forma de presentación del COVID19	◯ Asintomática<br>⊗ Sintomática
Gravedad OMS	◯ No clasificable<br>⊗ Leve<br>◯ Moderada<br>◯ Grave<br>◯ Critica
¿Cumple con los criterios para el síndrome inflamatorio multisistémico asociado con COVID?	◯ Si<br>⊗ No

Sistemas comprometidos	☐ Generales<br>☐ Pulmonares<br>☐ Cardiovasculares<br>☐ Neurológicos<br>☐ Gastrointestinales<br>☐ Renales<br>☒ Piel<br>☐ Otros<br>(Escoge los sistemas en los cuales se presentaron los síntomas del paciente)
¿Qué síntomas dermatológicos?	☐ lesiones acrales<br>☐ exantemas vesiculares<br>☐ erupciones urticariales<br>☒ exantemas maculopapulares<br>☐ lesiones livedoides/necróticas<br>(Escoja todos los que presentó el paciente)
Saturación O2 (SpO2) mas baja durante estancia	98<br>(0-100 %)
Fracción de Oxigeno (FiO2) para esa saturación.<br>(Solo en sistemas con FiO2 medida)	21<br>(21-100 %)

ONCOCOVID

Fecha de inicio de síntomas COVID	[*DATA REMOVED*]<br>(año-mes-día)
Fecha de toma de prueba positiva	[*DATA REMOVED*]<br>(año-mes-día)
Tiempo (días) entre inicio de síntomas y prueba positiva inicial	6
Tiempo (días) entre inicio de síntomas y admisión	3
Tipo de Prueba	⊗ RT-PCR<br>◯ Antigeno<br>◯ Anticuerpos
¿Tuvo más de una prueba positiva?	⊗ 1. Si<br>◯ 0. No
Tipos de prueba adicionales	☐ RT-PCR<br>☒ Antígeno<br>☐ Anticuerpos<br>☐ Otra
Antígeno Adicional	☒ Después de 10 días de la inicial<br>☐ En los primeros 10 días de la inicial

ONCOCOVID

¿Se detectaron positivos microbiológicos adicionales?	◯ Si<br>⊗ No
¿Se CULTIVARON positivos microbiológicos adicionales?	⊗ Si<br>◯ No
Sitio de cultivo	☒ Sangre<br>☐ Orina<br>☐ Traquea/Esputo<br>☐ Pleura<br>☐ LCR<br>☐ Tejido<br>☐ Otro
nombre de los aislamientos por cultivo	[*DATA REMOVED*]<br>(Escriba los nombres taxonómicos sin abreviaturas)

ONCOCOVID

En esta sección describa los laboratorios más anormales (PEORES) obtenidos durante la estancia en relación con su enfermedad aguda asociada a COVID.
(Si no se tomó el laboratorio deje vacío y marque el botón no se tomó).
Verifique que las unidades de medida coincidan con las solicitudes; de lo contrario, realice la conversión adecuada.

¿Se tomo datos de gases?	⊗ Si arteriales<br>◯ Si venosos<br>◯ No se tomó
pH	7.3<br>(Rango normal 7.35-7.45)
pCO2 (mmHg)	26.9<br>(Rango normal 35-45)
HCO3 (mmol/L)	21.1<br>(Rango normal 18-25)
PaO2 (mmHg)<br>Solo arterial	74.4<br>(Rango normal 60-100)
FiO2 (%)<br>Tomados en la muestra arterial reportada.	21<br>(21-100 %)
Lactato (mmol/L)	0.8<br>(Rango normal 0-2)
Trigliceridos (mg/dl)	____________________<br>(Rango normal 0-150)
	⊗ No se tomó
Lactato Deshidrogenasa [LDH] (U/L)	597<br>(Rango normal 50-150)
Nitrógeno Ureico [BUN] (mg/dL)	44<br>(Rango normal 7-20)
Creatinina (mg/dL)	0.3<br>(Rango normal 0.2-1.4)
Glucosa (mg/dl)	97<br>(Rango normal 60-140)
Hemoglobina [Hb] (g/dL)	6.8<br>(Rango normal 12-16)

Leucocitos totales (103/uL)	3.65<br>(Rango normal 4.5-12.0)
¿Presenta neutropenia severa (< 500)?	⊗ Si<br>◯ No
Fecha de neutropenia severa	[*DATA REMOVED*]
¿Presenta linfopenia severa (< 1000)?	◯ Si<br>⊗ No
Plaquetas (103/uL)	7<br>(Rango normal 150-400)
Proteina C reactiva (mg/dl)	26.7<br>(Rangos normales 0.1-0.5)
Transaminasa glutámico-oxalacética [TGO/AST] (U/L)	55
Transaminasa glutámico-pirúvica [TGP/ALT] (U/L)	17
Tiempo de protrombina [TP] (seg)	103.48
Índice internacional normalizado [INR]	2.9
Tiempo de tromboplastina parcial activada [TTPA/TTP] (seg)	45.3
Fibrinogeno (mg/dL)	1
Dimero D (mg/dL)	15.4<br>(Ej. 550)
Albumina (g/dl)	4.31<br>(Ej. 4.5)
Sodio (mmol/L)	127<br>(Ej. 135)
Potasio (mmol/L)	4.05<br>(Ej. 3.5)
Fosforo (mg/dL)	4.1<br>(Ej. 2.5)
Ácido úrico (mg/dL)	________________<br>(Ej. 6.2)
	⊗ No se tomó
Ferritina (ng/mL)	604<br>(Ej. 520)

Troponina I (ng/ml)

____
(Ej. 0.2)

⊗ No se tomó

ONCOCOVID

Ecocardiograma	⊗ Si<br>◯ No
Hallazgos (PEORES)<br>(describa los resultados mas anormales)	☒ Fracción de eyección<br>☐ Coronarias<br>☐ Pericardio<br>☐ Otro
Fracción de eyección (%)	44<br>(EJ: 45)
¿Se toma radiografía de tórax?	⊗ Si<br>◯ No
Radiografía de tórax (Peor)	☐ Compromiso de 1 cuadrante<br>☐ Compromiso de 2 cuadrantes<br>☐ Compromiso de 3 cuadrantes<br>☐ Compromiso de 4 cuadrantes<br>☐ Derrame Pleural<br>☐ Neumotórax<br>☒ Normal<br>☐ Otros
¿Se toma tomografía (TAC) de tórax?	◯ Si<br>⊗ No
¿Otra imagen con anomalías extrapulmonares?	⊗ Si<br>◯ No
¿Cuál imagen?	☒ Ultrasonido<br>☐ Doppler<br>☐ Resonancia<br>☒ Tomografía<br>☐ Otra
¿Qué órgano?	☐ Cerebro<br>☐ Riñón<br>☐ Hígado<br>☒ Otro
Hallazgos otra imagen	[*DATA REMOVED*]

ONCOCOVID

¿Presentación inicial (Debut)?	⊗ Si<br>◯ No
Tipo de neoplasia	⊗ Hematológico<br>◯ Tumor Sólido
Tipo hematológico	⊗ Leucemia linfoide aguda (LLA)<br>◯ Leucemia mieloide aguda (LMA)
¿Recibió quimioterapia para el tumor actual antes de esta infección?	◯ Si<br>⊗ No<br>◯ Desconocido
	⊗ Desconocido
Fase de quimioterapia	◯ Inducción<br>◯ Consolidación<br>◯ Mantenimiento<br>⊗ No aplica<br>◯ Desconocida
¿Recibió radioterapia en los últimos 30 días?	◯ Si<br>◯ No<br>⊗ Desconocido
¿Recibió terapia BIOLOGICA en los últimos 30 días?	◯ Si<br>◯ No<br>⊗ Desconocido
¿Recibió un trasplante de médula ósea?	◯ Si<br>◯ No<br>⊗ Desconocido

ONCOCOVID

Soporte respiratorio inicial (primer soporte utilizado al ingreso)	⊗ ninguno (Ambiente)<br>○ Sistemas de bajo flujo (Cánula, cámara cefálica, mascara libre)<br>○ Sistemas de alto flujo (Sistemas Venturi, Mascara con reservorio)<br>○ Cánula nasal de alto flujo<br>○ CPAP<br>○ Ventilación no invasiva (VNI)<br>○ Ventilación mecánica invasiva (IMV)
Soporte respiratorio máximo	○ ninguno (Ambiente)<br>○ Sistemas de bajo flujo (Cánula, cámara cefálica, mascara libre)<br>○ Sistemas de alto flujo (Sistemas Venturi, Mascara con reservorio)<br>○ Cánula nasal de alto flujo<br>○ CPAP<br>○ Ventilación no invasiva (VNI)<br>⊗ Ventilación mecánica invasiva (IMV)
¿Recibe corticoides para COVID?	⊗ Si<br>○ No
¿Qué corticoide?	☐ Dexametasona<br>☒ Prednisona<br>☒ Metilprednisolona<br>☐ Otro
¿en pulso? (dosis muy altas de corticoides)	○ Si<br>⊗ No
¿Recibió fármacos vasoactivos?	⊗ Si<br>○ No

Puntuación vasoactivo-inotrópica (vis)
(Sume todas las drogas usando la siguiente conversión)
10,000 x dosis de vasopresina (U / kg / min) +
100 x dosis de epinefrina (µg / kg / min) +
100 x dosis de noradrenalina (µg / kg / min) +
50 x dosis de levosimendan (µg / kg / min) +
25 x dosis de olprinona (µg / kg / min) +
20 x dosis de azul de metileno (mg / kg / h) +
10 x dosis de milrinona (µg / kg / min) +
10 x dosis de fenilefrina (µg / kg / min) +
10 x dosis de terlipresina (µg / min) +
0,25 x dosis de angiotensina II (ng / kg / min) +
Dosis de dobutamina (µg / kg / min) +
Dosis de dopamina (µg / kg / min¡ +
Dosis de enoximona (µg / kg / min)

Puntuación vasoactivo-inotrópica (vis)	20
Inmunoglobulina	◯ Si<br>⊗ No
Antibiótico	⊗ Si<br>◯ No
Anticoagulación	◯ Profiláctica<br>◯ Terapéutica<br>⊗ No

ONCOCOVID

Lugar de estancia<br>(elija todos los lugares donde estuvo su paciente)	☐ Consulta ambulatoria<br>☐ Consulta prioritaria<br>☒ Emergencias<br>☐ Planta pediátrica<br>☒ Cuidado intermedio<br>☒ Cuidado Intensivo<br>☐ Otra<br>(señale todas los servicios donde fue atendido el paciente en su centro por el cuadro actual)
Fecha Ingreso a UCI	[*DATA REMOVED*]
Fecha Egreso UCI	[*DATA REMOVED*]
Estancia UCI (días)	4
Fecha Egreso Hospitalaria	[*DATA REMOVED*]
Estancia Hospital (días)	60
Muerte hospitalaria	◯ Si<br>⊗ No
Durante la estadía, ¿desarrolló un cuadro de shock?	⊗ Si<br>◯ No
¿Qué tipo de shock?	☒ Septico<br>☐ Cardiogenico<br>☐ Obstructivo<br>☒ Vasoplejico<br>☐ Hipovolemico<br>(Puede escoger mas de uno si aplica)
Durante la estadía, ¿desarrolló un cuadro de síndrome hemo fagocítico/activación macrofágica?	◯ Si<br>⊗ No
Durante la estadía, ¿Fue llevado a cirugía?	⊗ Si<br>◯ No
¿Cuál cirugía y motivo?	[*DATA REMOVED*]
Durante la estadía, ¿desarrolló alguna difusión orgánica?	⊗ Si<br>◯ No
¿Qué disfunciones orgánicas?	☒ Cardiovascular<br>☒ Hematológico<br>☐ Hepático<br>☐ Neurológico<br>☒ Renal<br>☐ Respiratorio<br>(Puede escoger mas de uno si aplica)

ONCOCOVID

Esta sección busca datos faltantes, inconsistentes o erróneos para revisar. Cuando vea algún mensaje de modificación, revise la ficha correspondiente y márquela como no verificada al realizar la corrección para ser verificada nuevamente. Cuando se verifique la corrección, se marcará en esta sección como corregido y completo.

Concepto	⊗ Completo<br>○ Modificaciones<br>○ Excluir/borrar
Observaciones	[*DATA REMOVED*]
